# A Challenging Case of a Patient With Immune Thrombocytopenic Purpura on Eltrombopag Who Developed Atrial Fibrillation: An Anticoagulation Dilemma

**DOI:** 10.7759/cureus.35001

**Published:** 2023-02-15

**Authors:** Ahmed K Yasin, Mohammad Abu-Tineh, Awni Alshurafa, Khalid Ahmed, Mohammed Abdulgayoom, Mohammad Afana, Mohamed A Yassin

**Affiliations:** 1 Department of Internal Medicine, Hamad Medical Corporation, Doha, QAT; 2 Department of Medical Oncology, Hematology and Bone Marrow Transplant Section, National Center for Cancer Care and Research, Hamad Medical Corporation, Doha, QAT

**Keywords:** hematology, atrial fibrillation, immune thrombocytopenic purpura, anticoagulation, cardiovascular disorders

## Abstract

A 61-year-old female, who was a known case of immune thrombocytopenic purpura (ITP) on eltrombopag, was admitted for atrial fibrillation (AF). Labs showed a platelet count of 116 × 10^3^/µL. AF reverted to sinus rhythm by cardioversion. Therapeutic enoxaparin was started for two days. She was discharged on dabigatran for four weeks. The choice of anticoagulation in these cases (ITP and AF) is not straightforward and needs further research.

## Introduction

Immune thrombocytopenic purpura (ITP) is defined as an autoimmune disorder characterized by a low platelet count (<100 × 10^9^/L) with a variably increased risk of bleeding [1]. ITP may occur in the absence of an apparent predisposing etiology (primary ITP) or as a sequela of an associated condition (secondary ITP) [2]. ITP is a diagnosis of exclusion. Usually, it presents with sudden-onset petechial rash and/or bruising. Secondary causes should be explored in history such as systemic symptoms (weight loss, fever, anorexia), recent medications, viral illnesses, family history of bleeding disorders, rheumatological diseases, or liver diseases. In most cases, the only abnormality seen in blood investigations is a low platelet count. On peripheral smears, cells usually have a normal appearance with a low platelet count [3].

Eltrombopag and rituximab are considered second-line therapy for patients with ITP who do not respond to first-line management (corticosteroids). Eltrombopag is a thrombopoietin receptor agonist (TPO-RA) approved for the treatment of patients with chronic ITP who are older than one year. Eltrombopag increases platelet production by binding the transmembrane domain of the TPO-RA and activating the proliferation of megakaryocytes from bone marrow progenitors [4]. Eltrombopag increases platelet counts in most patients with ITP to adequate levels. It is well tolerated and safe, and important adverse events such as thrombosis or bone marrow fibrosis are infrequent [4].

ITP has been reported to be associated with thrombotic events. A previously reported univariate analysis showed that smoking, hypertension, male gender, a history of thrombosis, and atrial fibrillation (AF) were significantly associated with the occurrence of thrombosis [5]. Many patients with ITP are old and have comorbidities including AF. Guidelines do not provide clear guidance for anticoagulation in these patients. Anticoagulation in patients with ITP and AF is a clinical challenge because it is usually contraindicated when the platelet count is less than 50,000 [6].

## Case presentation

**Figure 1:**
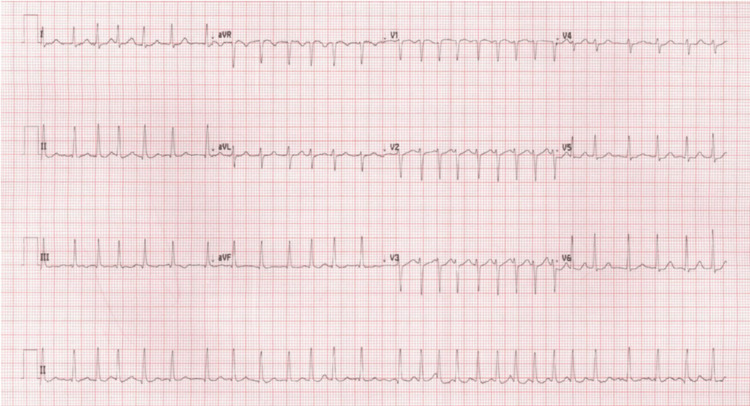
Electrocardiogram at presentation.

A 61-year-old female, who was a known case of ITP on eltrombopag 50 mg daily for four years and had failed steroids as first-line and rituximab as second-line therapy, presented to the emergency department complaining of chest pain and palpitations started at midnight. She denied nausea, vomiting, pain radiating to the shoulders, shortness of breath, dizziness, or other symptoms. Her heart rate was 168 beats per minute, and the electrocardiogram (ECG) showed AF with a rapid ventricular response (Figure 1). Her blood pressure was stable, and she was on room air with an oxygen saturation of 99%.

Lab results on admission are shown in Table 1.

**Table 1:** Laboratory results of the patient.

Lab	Result	Reference range
White blood cells	9.7 × 10^3^/µL	4.0–10.0
Hemoglobin	12.2 g/dL	12.0–15.0
Platelets	116 × 10^3^/µL	150–400
Prothrombin time	11.1 seconds	9.7–11.8
Partial thromboplastin time	26.7 seconds	24.6–31.2
International normalized ratio	1	NA
Sodium	144 mmol/L	133–146
Potassium	4.1 mmol/L	3.5–5.3
Adjusted calcium	2.45 mmol/L	2.20–2.60
Phosphorus	1.30 mmol/L	0.80–1.50
Magnesium	0.85 mmol/L	0.70–1.00
Creatinine	66 µmol/L (0.75 mg/dL)	44–80 (0.74–1.35)
Urea	5.1 mmol/L	2.5–7.8
Alanine transaminase	13 U/L	0–33
Aspartate transaminase	19 U/L	0–32
Lactate dehydrogenase	249 U/L	135–214
Troponin-T	5 ng/L	3–10
Iron	11 µmol/L	6–35
Ferritin	13.9 µg/L	18–340
Total iron-binding capacity	83 µmol/L	45–80
Transferrin	3.3 g/L	2.0–3.6
Iron saturation	13%	15–45
Vitamin B12	415 pmol/L	145–596
C-reactive protein	1 mg/L	0.0–5.0

A chest X-ray showed prominent broncho-vascular markings. Transthoracic echocardiography showed normal left ventricular function, ejection fraction of 57%, no regional wall abnormalities, severely dilated left atrium (diameter 4.4 cm), and no thrombus in the left atrium.

She was admitted to the cardiology ward, given an intravenous amiodarone bolus of 300 mg, and then kept on an amiodarone infusion of 900 mg over 24 hours. Because AF persisted, she was given again another amiodarone infusion at the same dose for another 24 hours. After that, AF was still present, so transesophageal echocardiography was done, which showed no thrombi. Subsequently, electrical cardioversion was done which reverted the AF to sinus rhythm.

Therapeutic enoxaparin was started initially by the cardiology team as platelets were more than 50 after discussion with the hematology team.

CHA₂DS₂-VASc Score was 1, HAS-BLED score was 1, and there was no history of severe bleeding with World Health Organization grade 3 or 4. Hence, there was no need for lifelong anticoagulants; however, after cardioversion, the patient needed to be on anticoagulation for four weeks. The case was discussed with hematology again, and they agreed that the patient can be started on non-vitamin K antagonist oral anticoagulants (NOAC), but needed to get her complete blood count (CBC) and platelets checked weekly. Moreover, if platelets dropped below 50,000, the therapy had to be stopped.

During her stay, a CT coronary angiography was done which showed normal coronaries. She was also kept on a beta-blocker (metoprolol) with a gradually increasing dose.

The final plan from hematology upon discharge was to continue eltrombopag 75 mg daily, to continue dabigatran 110 mg twice daily for four weeks, and no contraindication of anticoagulation as long as platelets were more than 50,000. The patient was discharged with good general condition and stable vital signs. She is being followed up in the clinic as an outpatient and is doing well with no complications.

## Discussion

Evidence to guide appropriate anticoagulation therapy in the setting of ITP and AF is very limited. Data from cohort studies suggest that a reduced-dose direct oral anticoagulant (rivaroxaban 15 mg once daily, dabigatran 110 mg twice daily (bid), or apixaban 2.5 mg bid) is effective and safe with mild thrombocytopenia (platelet count between 50,000 and 100,000/µL) [7].

In another study, a comparison between NOACs and warfarin demonstrated a significantly better clinical outcome for patients on NOACs in both the low platelet group (lower mortality rates) and normal platelet group (lower mortality, transient ischemic attacks, cerebrovascular accidents, and systemic emboli rates) [8]. From these data, it was decided to keep the patient on dabigatran 110 mg twice daily for four weeks as long as the platelet count was more than 50,000/µL.

For patients with ITP on eltrombopag, CBC with differential and platelet count should be done weekly at initiation and during dosage titration, followed by monthly when stable. Liver functions tests, including alanine transaminase, aspartate aminotransferase, and bilirubin, should be checked at baseline, every two weeks during dosage titration, and then monthly after a stable dose is achieved [9].

Eltrombopag is associated with an infrequent increase in thromboembolic events [10,11]. In the EXTEND trial, 11 out of 299 patients (4%) developed thromboembolic events [4]. NOACs in patients with AF and thrombocytopenia are associated with an increased risk of bleeding [7]. Hence, clinical follow-up of patients is crucial with frequent questions about bleeding/thrombosis symptoms and signs. In one study, the most common adverse events were anemia, upper respiratory tract infection, and cough, hence, it is advisable to ask patients about these symptoms [12-14].

This case report highlights the importance of further studies and trials regarding the choice of appropriate anticoagulation in patients with ITP and AF and more specifically those on eltrombopag. It also confirms that in an ITP patient who is at low bleeding risk by eltrombopag treatment, the accompanying AF can be treated by NOACs without severe bleeding or thromboembolic complications at least for a short period of anticoagulation.

## Conclusions

In patients with ITP who are treated with eltrombopag and then develop AF, the choice of anticoagulation is a clinical dilemma due to the conflicting risks of thrombosis and bleeding. Clinical data are limited but studies have shown that NOACs are safe and result in better outcomes than warfarin. Patients should be closely followed up and monitored.
